# Impact of extreme weather events on food security among older people: a systematic review

**DOI:** 10.1007/s40520-025-03050-3

**Published:** 2025-04-30

**Authors:** Daniele Nucci, Flavia Pennisi, Antonio Pinto, Emanuele De Ponti, Giovanni Emanuele Ricciardi, Carlo Signorelli, Nicola Veronese, Alberto Castagna, Stefania Maggi, Chiara Cadeddu, Vincenza Gianfredi

**Affiliations:** 1 Struttura Semplice Dipartimentale Igiene Alimenti e Nutrizione, Dipartimento di Igiene e Prevenzione Sanitaria, Agenzia di Tutela della Salute (ATS) Brescia, Via Duca degli Abruzzi, 15, 25124 Brescia, Italy; 2 PhD National Program in One Health Approaches to Infectious Diseases and Life Science Research Department of Public Health, Experimental and Forensic Medicine, University of Pavia, 27100 Pavia, Italy; 3 Faculty of Medicine, University Vita-Salute San Raffaele, 20132 Milan, Italy; 4 Faculty of Medicine, Saint Camillus International University of Health Sciences, Rome, Italy; 5 Department of Primary Care, Health District of Soverato, Azienda Sanitaria Provinciale, Catanzaro, Italy; 6 National Research Council (CNR), Aging Section, Padova, Italy; 7 Erasmus School of Health Policy and Management, Erasmus University Rotterdam, Rotterdam, Netherlands; 8 Department of Biomedical Sciences for Health, University of Milan, via Pascal, 36, 20133 Milan, Italy

**Keywords:** Climate change, Food insecurity, Malnutrition, Elderly populations, Extreme weather events, Public health

## Abstract

**Background:**

Climate change has intensified the frequency and severity of extreme weather events, disproportionately affecting vulnerable populations, including older people for which the literature is still limited. This systematic review investigated the impact of extreme weather events on malnutrition and food security among individuals aged 60 and older.

**Methods:**

A systematic search of PubMed/MEDLINE, Scopus, and Web of Science was conducted without restrictions (October 2024), and following PRISMA guidelines. Observational studies examining older adults exposed to extreme weather events (e.g., droughts, floods, heatwaves, hurricanes) and their effects on malnutrition or food security were included. The Newcastle-Ottawa Scale assessed study quality. Protocol was registered in PROSPERO (ID: CRD42024596910).

**Results:**

From 1,709 articles, six observational studies involving 265,000 participants (aged 60 years and over) were included. These studies spanned multiple geographies, with a concentration in the United States. Findings revealed a dual impact: while some studies reported protective factors, such as social support and economic stability, others highlighted increased malnutrition risk due to disrupted food supply, economic hardship, and inadequate adaptive responses. Heterogeneity in study designs, exposure definitions, and outcome measures limited comparability.

**Conclusion:**

Extreme weather events significantly impact malnutrition and food security among older adults, with outcomes influenced by socio-economic and geographical factors. Further longitudinal studies are needed to clarify causal pathways and inform targeted public health interventions to enhance resilience in aging populations.

## Introduction

Climate change represents an urgent and complex challenge for public health. As estimated by the World Health Organization (WHO) between 2030 and 2050, 250,000 additional deaths per year could be attributable to climate change [1].

Climate change is primarily driven by the rise in global temperatures, a consequence of increased greenhouse gas emissions largely caused by human activities [2]; and it is characterized by extreme weather events such as heat waves, storms, wildfires, floods, and droughts. These events are increasing in frequency and intensity and pose the global food system under pressure. For instance, climate change exacerbates the spread of infectious diseases, including water-borne, food-borne, and vector-borne diseases, as changing temperatures and precipitation patterns create favourable conditions for pathogens and their vectors [3]. Furthermore, extreme weather events can disrupt agricultural systems, compromising the ability of individuals - especially in low and middle income countries- to access nutritious and safe diets [4, 5], leading to reduced crop yields, food insecurity, and malnutrition [6]. Food insecurity and malnutrition remain major global public health concerns, with climate change acting as an increasingly critical driver. According to the Food and Agriculture Organization (FAO), approximately 2.4 billion people experienced moderate or severe food insecurity in 2022, with about 735 million suffering from chronic hunger—a figure that has worsened due to conflict, socioeconomic inequality, and climate-related shocks [7]. These nutritional deficiencies can have cascading effects on health, particularly among vulnerable populations, such as the elderly, children, and those with pre-existing health conditions [8]. While the impact of climate change on food security has been extensively discussed, specific attention to older adults remains limited, despite this group’s rapidly growing numbers and heightened vulnerability.

Globally, the older population is growing, reflecting a progressive aging of society [9]. Older adults face unique physiological, social, and economic challenges—including multimorbidity, reduced biological resilience, limited mobility, and fixed incomes—that may reduce their ability to adapt to or recover from environmental, placing older individuals in a state of vulnerability [10]. This vulnerability may be further exacerbated by climate change, as the aging body typically has reduced biological resilience [11]. Additionally, older adults may face greater challenges in coping with the catastrophic effects of extreme weather events, such as heatwaves, floods, or storms, further amplifying their risk of adverse health outcomes. In this framework, the intersection of climate change and population aging presents a critical issue of paramount importance. However, the studies currently available on the topic are limited, and the results are conflicting. In light of this, this systematic review aimed to explore the impact of extreme weather events on malnutrition and food security among individuals aged 60 and older. This systematic review aims to fill a critical gap by investigating the impact of extreme weather events on malnutrition and food security among individuals aged 60 and older. To our knowledge, this is the first review that specifically focuses on this intersection—integrating diverse indicators (e.g., BMI, dietary intake, hospitalization data, and validated food insecurity scales) and drawing comparisons across countries with varying income levels and disaster response capacities.

By analyzing findings across diverse geographic and socio-economic contexts, this study seeks to provide insights into the scale of the problem, identify key risk factors, and inform targeted policy interventions to enhance resilience and adaptive capacity among aging populations.

## Methods

This systematic review was conducted in accordance with the guidelines of the Preferred Reporting Items for Systematic Reviews and Meta-Analyses (PRISMA) [12] and was pre-registered in PROSPERO, the international prospective register of systematic reviews, prior to initiation (ID: CRD42024596910).

### Information sources and search strategy

A comprehensive search of PubMed/MEDLINE, Scopus, and Web of Science databases was conducted without time or language restrictions. The search strategy included combinations of keywords and Medical Subject Headings (MeSH) terms related to climate change (and sinonyms), malnutrition and food security (and sinonyms), and older adults (and sinonyms). Specific search strings were tailored to each database. The search strings are available in supplementary Table 1.

### Eligibility criteria

We included studies focusing on older adults (aged 60 and above) in regions affected by extreme events resulting from climate change, such as droughts, floods, heatwaves, and hurricanes. Studies were required to explicitly assess the impact of these events on malnutrition or food security status. Observational studies, published in English, as peer-reviewed articles in international scientific journals were eligible for inclusion. Studies were excluded if they focused on populations other than older adults, did not link climate change to nutrition or food security, or presented outcomes unrelated to malnutrition or food security (e.g., dietary habits or nutrient deficiencies). The inclusion/exclusion criteria have been defined according to the Population, Exposure, Comparison, Outcome and Study design (PECOS) acronym, as reported in Table 1.

**Table 1:** Details of inclusion/exclusion criteria, according to population, exposure, comparison, outcome and study design (PECOS) acronym

	Inclusion Criteria	Exclusion Criteria
Population	Older adults (60+) in climate-affected regions, directly impacted by climate change	Non-older adults, general population, or studies without specific data on older adults
Exposure	Extreme events caused by climate change (e.g., droughts, floods, heat waves, hurricanes)	Studies not linking climate change to food security or nutrition
Comparator	Time periods (pre- vs. post) or regions without significant extreme events secondary to climate change effect	Comparators not relevant to climate change’s effects on food security or nutrition
Outcomes	Malnutrition and food security status	outcomes not related to nutrition or food security, including dietary habits, food intake or micro-macro nutrients deficiency
Type of studies	Original, observational study (including cross-sectional, case-control, or cohort both prospective and retrospective studies), published as peer-reviewed articles in international scientific journals	Intervention, not original (reviews with or without meta-analysis), not performed among humans, not observational (as for instance trials), not published as peer-reviewed articles in international scientific journals (book, book chapter, thesis), no full-text papers (abstract, conference paper, letter, commentary, note)
Language	English	Different than English
Time filter	None	None

### Study selection

Two independent reviewers (FP, EDP) used Rayyan to screen the titles and abstracts of retrieved articles for eligibility. Full-text articles were obtained for studies meeting the inclusion criteria, and their eligibility was confirmed by assessing adherence to the defined criteria. Any discrepancies were resolved by consensus or through consultation with a third reviewer (AP).

### Data extraction

Two reviewers independently extracted data using a standardized form, pre-piloted on 2 randomly selected included studies. Extracted information included:

- Study characteristics: Author(s), year of publication, country, study design, sample size, study name (if applicable), and study period.
- Population characteristics: Age range, gender distribution, geographic region, and number of participants exposed and not exposed to extreme events.
- Exposure: Type of extreme event (e.g., floods, droughts) and type of comparison (pre-post).
- Outcomes: Measures of malnutrition and food security, tools used for assessment (e.g., Mini Nutritional Assessment, Body Mass Index (any), Food Insecurity Experience Scale), and maximally adjusted effect size measurements with corresponding 95% confidence intervals.
- Additional study information: Funding sources and conflict of interest declarations.

Any missing data were requested directly from the corresponding authors of the included studies.

### Risk of bias assessment

The quality of the included studies was independently evaluated by two blinded raters (AP, EDP) using the Newcastle-Ottawa Scale (NOS) for non-randomized studies, as proposed by GA Wells et al. [13] This tool utilizes two separate checklists for case-control and cohort studies, assessing three key domains: the selection of study groups, their comparability, and the ascertainment of outcomes. Each study was rated on a scale from 0 to 9 stars, reflecting its methodological rigor. For cross-sectional studies, we applied an adapted version of the NOS checklist by R Herzog et al. [14], which evaluates methodological quality on a scale from 0 to 10 stars. Based on their adherence to the checklist criteria, the included studies were categorized as high quality (≥ 8), moderate quality (5–7), and low quality (≤ 4).

## Results

### Literature search

**Fig. 1:**
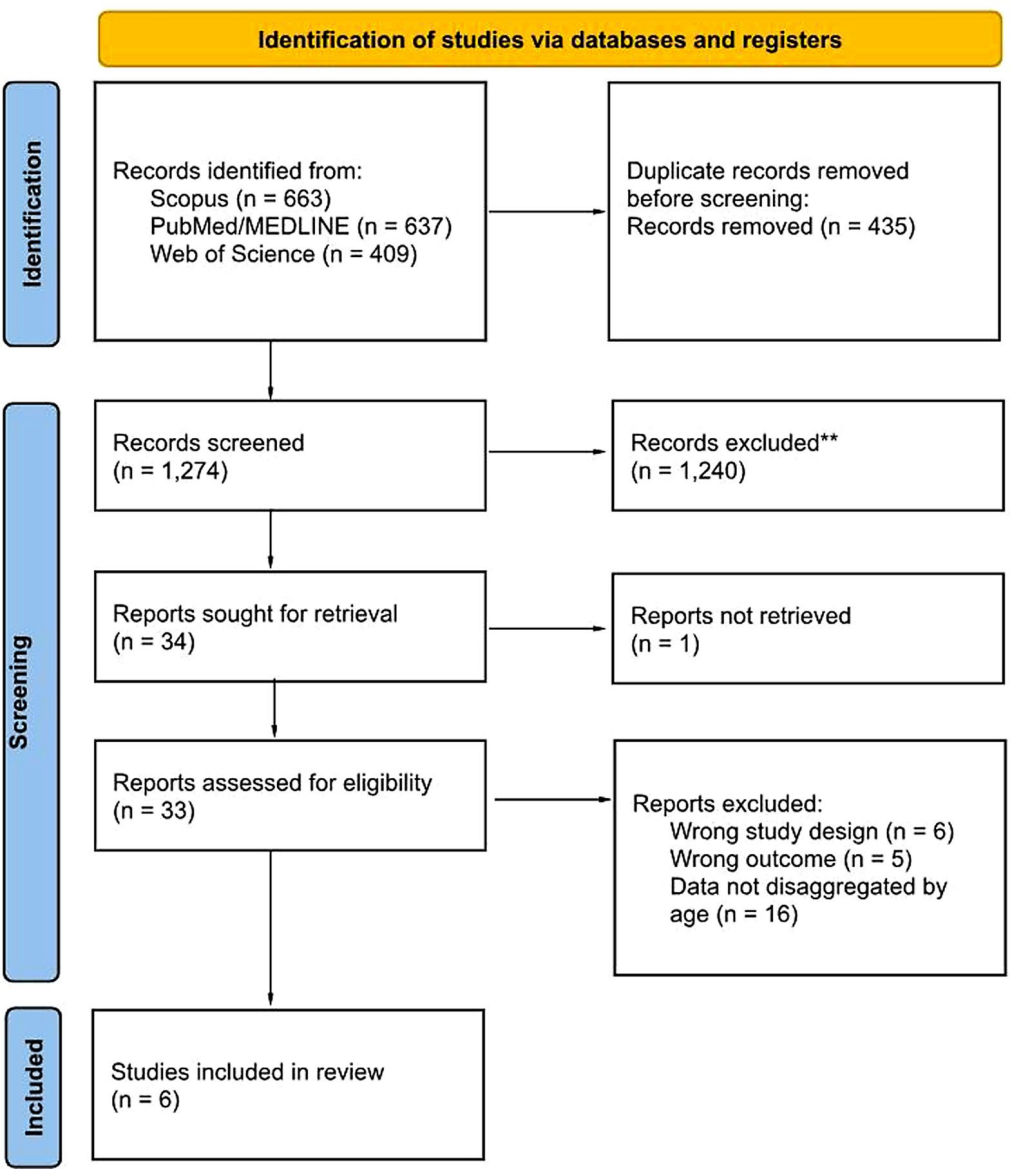
Flow diagram depicting the selection process

A total of 1,709 records were identified through searches in Scopus (*n* = 663), PubMed/MEDLINE (*n* = 637), and Web of Science (*n* = 409) databases. Following the initial removal of duplicates (*n* = 435), a total of 1,274 records underwent screening based on title and abstract. Subsequently, 1,240 records were eliminated due to non-original content and focus on different topics, resulting in 34 records deemed eligible for inclusion. One article lacked full-text availability. Following the full text assessment, 27 records were excluded, including 6 records. The most common reason for exclusion was the data not disaggregated by age (*n* = 16), wrong study designs (*n* = 5), followed by wrong outcome (*n* = 5). The study selection process is visually represented in Fig. 1.

### Descriptive characteristics of included studies

The systematic review comprised six studies [15–20], all published after the year 2009. The geographic distribution is enough concentrated, with the highest contribution from the USA (*n* = 3 studies) [16–18], and one study from Brazil [20], China [19] and India [15]. Most studies (83.3%, *n* = 5) had a national approach [15–17, 19, 20], while only one was regionally focused [18]. Four studies obtained data only from surveys [15–18], conducted with households severely impacted by extreme weather events, instead of one that used both from a survey and from a climate research database (temperature and precipitation) [19], while one only used hospitalization data and daily meteorological data (like temperature, humidity) [20]. The samples from all studies stratify patients by age groups, and all include the over-60 age group, which was the focus of our study. Five articles reported a sample with a predominance of females, reaching a peak of 61.87% [15–19], while only one study had a male dominance (55.5%) in its sample [20].

The majority of the studies (50.0%, *n* = 3) were on populations affected by hurricanes [16–18], two concerned temperatures (heat exposure, hot season with temperature increase) and precipitation anomalies [19, 20], and one was about drought [15]. Due to the limited number of included studies and the substantial heterogeneity in outcome measurements, a meta-analysis was not conducted (Table 2).

**Table 2:** Summary of study characteristics: design, population and exposure details

Author, year (Ref)	Country	Level	Study period	Study type	Data sources	Follow-up time	Age range	Gender distribution (female)	Population characteristics at background	Climate change-affected regions	Type of extreme event
Arlappa N, 2009 [15]	India	N	May and June 2003	CSS	Survey	NA	≥ 60	58.3%	Older adults in rural communities, with high vulnerability to drought.	9 states of India declared severely drought-affected	Drought
Clay LA, 2018a [16]	USA	N	2006–2010	CS	Survey	5 years after Hurricane Katrina	≥ 66	61.87%	Multiethnic, economically stable households severely impacted by Hurricane	Gulf Coast, heavily impacted by Hurricane Katrina	Hurricane (Hurricane Katrina)
Clay LA, 2018b [17]	USA	N	2007–2010	LS	Surveys conducted with households severely impacted by Hurricane Katrina	5 years (data collected from 2007–2010)	≥ 66 years	60.7% female; the gender distribution for the 66 + age group not explicitly stated	Racial/Ethnic Distribution: 51.5% Black, 43% White, 2.7% Latino, 2.8% Other; Gender: 60.7% female (general population, assumed similar for 66+); Income: <$10K: 28.7%, $10K–$20K: 31.5%; Employment: 40% employed; Physical Health: 51.7% poor physical health; Mental Health: 38.5% poor mental health; Disability: 20.5% with disability; Social Support: 15.3% low social support	Gulf Coast (Louisiana and Mississippi, heavily impacted by Hurricane Katrina)	Hurricane (Hurricane Katrina)
Clay LA, 2020 [18]	USA	R	12–15 months post-Hurricane Harvey (Survey conducted in 2018)	CSS	Web Survey	12–15 months after Hurricane Harvey (cross-sectional survey, not a cohort study)	≥ 65 years: Retirement age (15.3% of the sample)	57.4% female; the gender distribution for 65 + age not separately listed	Racial/Ethnic Distribution (> 60 years): 51.5% Black, 43% White, 2.7% Latino, 2.8% Other; Income: <$15K: 14.2%, $15K–$24.9 K: 12.2%, $25K–$34.9 K: 11.0%, $35K–$49.9 K: 14.8%, $50K–$74.9 K: 20.0%, $75K–$99.9 K: 13.1%, $100K+: 14.7%; Employment: 10.6% unemployed; Physical Health: 71.5%; Mental Health: 57.2%; Social Support: 76.4% high social support	41 countries in Texas affected by Hurricane Harvey	Hurricane (Hurricane Harvey)
Mueller V, 2018 [19]	China	N	Multiple waves from 1989 to 2011	LS	CHNS (1989–2011). Temperature and precipitation extracted from the CRUTS	NA	≥ 60	51.2% female	Rural and urban adult population in China, representing a variety of socioeconomic backgrounds	9 provinces across China (Heilongjiang, Jiangsu, Shangdong, Henan, Hubei, Hunan, Guangxi, Guizhou, and Liaoning)	Climate variability is measured as z-scores: deviations from the 1981–2010 mean, divided by the standard deviation.
Xu RB, 2019 [20]	Brazil	N	1 January 2000–31 December 2015	CCS	Hospitalization data from the BUHS and daily meteorological data (temperature, humidity)	NA	≥ 60	44.5% female	Total sample: Median Age: 57.9 years (IQR: 34.9–75.1 years)	Brazil, with significant regional variations in heat exposure and climate conditions	Heat exposure (hot season with temperature increase)

BUHS = Brazilian Unified Health System, CCS = Case-Crossover Study, CHNS = China Health and Nutrition Survey, CRUTS = Climate Research Unit’s Time Series, CS = Cohort Study, CSS = Cross-Sectional Study, IQR = Interquartile Range, LS = Longitudinal Study, N = National, R = Regional, USA = United States of America

### Malnutrition measures and comparison

Table 3 presents information on our included studies, addressing malnutrition and food insecurity among elderly individuals. The 6 included studies encompassed a total of about 265 thousand patients. Malnutrition, potentially present in the individuals included in the analysis following an extreme weather event, was assessed in three studies through food insecurity measures [16–18], in one study by evaluating undernutrition [20], in one study using BMI alone [15], and in another study using both BMI and daily caloric, protein, and fat intake [19]. To assess the association between natural disaster resulting from severe weather events and malnutrition, the studies employed various types of comparisons: three studies evaluated conditions before and after the event [15–17], two studies compared exposure days to control days [19, 20], while one study did not perform any comparison, reporting only post-event data following the extreme weather event [18]. Additionally, the table indicates that three of the included studies received funding to support their research [18–20], whereas none of the studies reported any conflicts of interest.

### Extreme weather event and positive impact on nutrition/food security

The three studies conducted by Clay et al. focus exclusively on the United States (USA) and analyze the effects of hurricanes, which are high-impact, short-duration extreme weather events [16–18]. The findings indicate that, in this context, older adults are less likely to experience food insecurity compared to younger age groups. The odds ratios show significantly reduced risks: Clay (2018a) reports an OR = 0.8 (95% CI: 0.35–1.81), Clay (2018b) an OR = 0.90 (95% CI: 0.83–0.98), and Clay (2020) an OR = 0.33 (95% CI: 0.19–0.59). This protective effect is attributed not only to factors such as greater resource availability and an effective national response to extreme events but also to social support and health policy interventions, which act as a safety net and potentially mitigate the risk among older adults. Furthermore, the lower impact observed in older adults may be linked to the economic stability provided by fixed incomes, such as pensions, making them less vulnerable to the economic consequences of climatic events. This contrasts with younger population groups, often of working age, who are more exposed to economic instability caused by events like hurricanes. All three studies aligned with Food and Nutrition Service criteria developed by the United States Department of Agriculture (USDA) to assess food insecurity [21], highlighting how the U.S. context, combined with the short duration of these climatic events, contributes to a lesser impact on malnutrition among older adults.

### Extreme weather event and negative impact on nutrition/food security

In contrast, the data from Arlappa N., Mueller V., and Xu R.B. demonstrate greater vulnerability among older adults to malnutrition in contexts characterized by prolonged extreme weather events, such as extended droughts, thermal anomalies, and precipitation variability [15, 19, 20]. The observed effects in these studies stem from extended observation periods, which are essential to capture the long-term impact on individuals aged over 60. Notably, two of the three studies report observation periods of 22 years [19] and 15 years [20], which are considerably longer than the periods covered in the studies that identified a protective association.

Arlappa N., examining the impact of drought in India, highlights a high prevalence of chronic energy deficiency (CED), with 51.1% of elderly men and 48.5% of elderly women exhibiting a BMI < 18.5 [15]. Mueller V., in China, finds that significant thermal anomalies increase the incidence of underweight among older adults (coefficient for BMI < 18.5: 0.011 per unit increase in temperature) [19]. Xu R.B., analysing hospitalizations in Brazil, reports that each 1 °C rise in daily mean temperature is associated with an increased risk of hospitalization for protein-energy malnutrition (OR = 1.022, 95% CI: 1.013–1.031) [20]. These studies, conducted in middle-income countries (MICs) namely India, China, and Brazil, suggest that the prolonged duration of extreme weather events, coupled with limited economic and infrastructural response capacities, amplifies the negative impact on older adults, rendering them more vulnerable compared to the contexts analysed in the United States.

**Table 3:** Key findings from included studies

Author, year (Ref)	Sample size	Type of comparison (pre-post or regions)	Measures of malnutrition	Maximally adjusted effect size measurements	Conclusions	Funds / CoI
Arlappa N, 2009 [15]	3,147 elderly adults (> 60 years and older) from 2,628 households in 190 villages	Drought period vs. non-drought period	BMI	BMI (male) mean: 18.8; BMI (female) mean: 19.1; BMI (male) distribution: <18.5 (51.1%), 18.5–25.0 (45.5%), ≥ 25.0 (3.4%); BMI (female) distribution: <18.5 (48.5%), 18.5–25.0 (44.4%), ≥ 25.0 (7.0%); NON DROUGHT PERIOD (originally published in another paper): BMI (male) distribution: <18.5 (53.5%), 18.5–25.0 (42.3%), ≥ 25.0 (4.2%); BMI (female) distribution: <18.5 (49.4%), 18.5–25.0 (42.9%), ≥ 25.0 (7.7%)	The prevalence of CED was a “very high” public health problem among older adults in drought-affected areas. The prevalence of CED was higher in the most advanced age groups. Undernutrition was more common among older persons belonging to low socioeconomic groups and those from below poverty line households	No / No
Clay LA, 2018a [16]	Total: 737 respondents; >60 years: 110 respondents	Pre-post (food insecurity before and 5 years after the disaster)	Food Insecurity: (respondents categorized as food insecure or food secure based on the frequency of not having enough money for food in the past 3 months)	Age 66 + years: OR 0.8 (95% CI 0.35, 1.81), indicating a lower likelihood of food insecurity compared to the 18–34 age group	Social support, self-efficacy, and community sense protected against food insecurity, which was influenced by economic stability, physical and mental health, and social factors.	No / No
Clay LA, 2018b [17]	Total: 683 households; >60 years: 125	Pre-post (food insecurity measured over a 5-year period post-disaster)	Food Insecurity: (respondents categorized as food insecure or food secure based on the frequency of not having enough money for food in the past 3 months)	Age 66 + years: OR 0.90 (95% CI 0.83, 0.98), indicating a lower likelihood of food insecurity compared to the 18–34 age group	66 + age group was protective against food insecurity, along with higher income and being partnered. Risk factors included poor physical health, mental health, low social support, and being female	No / No
Clay LA, 2020 [18]	Total: 1002; >60 years: 153	Post-event (food insecurity measured after Hurricane Harvey)	Food insecurity was assessed with a validated two-item food security screener: “We have worried whether our food would run out before we got money to buy more.” “The food we bought just didn’t last and we didn’t have money to get more.” Respondents answering “often true” or “sometimes true” to either statement were categorized as food insecure.	Age 65 + years: OR 0.33 (95% CI 0.19, 0.59) compared to 18–44 years, indicating that individuals aged 65 + were 67% less likely to report food insecurity than those in the 18–44 age group	Protective Factors for 65 + years: older age, better physical and mental health, greater income, and high social support were found to be protective against food insecurity. Risk Factors: non-white race, economic instability, major home damage, relocation, and community-based assistance, due to poverty, were found to increase the risk of food insecurity	Yes / No
Mueller V, 2018 [19]	20,990 individuals; >60 years: 21.3%	Comparison between days of extreme heat (exposure days) and normal temperature days (control days) within the same individual	BMI, daily caloric intake (kcal), daily fat intake (grams), daily protein intake (grams)	BMI: Temperature Anomaly: -0.056 (95% CI -0.140, 0.028), Precipitation Anomaly: +0.029 (95% CI -0.046, 0.104); Underweight Indicator (BMI < 18.5): Temperature Anomaly: +0.011 (95% CI 0.003, 0.019), Precipitation Anomaly: -0.004 (95% CI -0.010, 0.002); Daily Caloric Intake (kcal): Temperature Anomaly: -36.855 (95% CI -95.679, 21.969), Precipitation Anomaly: +31.115 (95% CI -15.895, 78.125); Daily Fat Intake (grams): Temperature Anomaly: -0.337 (95% CI -3.541, 2.867), Precipitation Anomaly: +1.004 (95% CI -1.304, 3.312); Daily Protein Intake (grams): Temperature Anomaly: -0.769 (95% CI -2.695, 1.157), Precipitation Anomaly: +0.524 (95% CI -0.993, 2.041)	Temperature anomalies significantly increase the incidence of underweight in adults over 60, while precipitation anomalies have minimal impact. Dietary intake changes due to these anomalies are negligible. A prediction model indicates that extreme heat and drought conditions (temperature z-score = 2, precipitation z-score = -2) could raise the probability of underweight in elderly individuals by 28%. For China’s elderly population in 2010 (178 million), this scenario translates to an estimated additional 5.9 million cases of malnutrition.	Supported by Arizona State University and the International Food Policy Research Institute / No
Xu RB, 2019 [20]	238,320 hospitalizations, the specific number for > 60 years hospitalizations is not separated out	Comparison of heat exposure during hospitalization days and control days	Undernutrition: categorized as severe PEM, moderate PEM, mild PEM, retarded development, and unspecified PEM	OR for hospitalization due to undernutrition with every 1 °C increase in daily mean temperature: 60–79 years: OR = 1.022 (95% CI 1.013–1.031), *p* < 0.001 AF for heat exposure in the elderly (60–79 years): 12.6% of undernutrition hospitalizations in the group were attributed to heat exposure (95% CI: -0.3–23.0%)	Heat exposure substantially raises the risk of hospitalization for undernutrition in elderly individuals, with 12.6% of hospitalizations in the 60–79 age group attributed to heat exposure. Older adults are more vulnerable than younger groups, exhibiting a significantly higher odds ratio for hospitalization.	Funded by the Australian National Health and Medical Research Council and China Scholarship Council / No

AF = Attributable Fraction, BMI = Body Mass Index, CED = Chronic Energy Deficiency, CoI = Conflict of interest, OR = Odds Ratio, PEM = Protein-Energy Malnutrition, USDA = United States Department of Agriculture

### Quality assessment

**Fig. 2:**
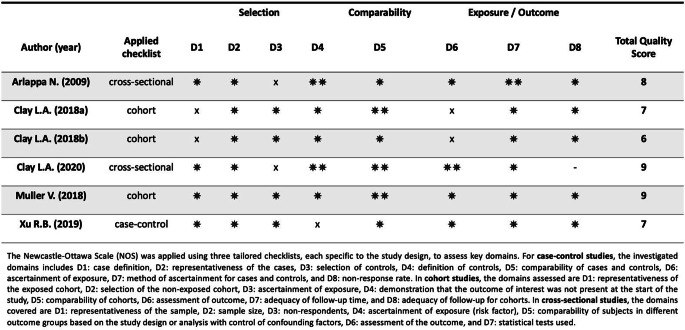
Quality assessment of the studies

Figure 2 presents a summary of the quality assessment results for the included studies, detailing the performance across each domain of the three NOS checklists which were employed. Based on the checklists criteria, three studies achieved a high-quality rating (≥ 8 stars) [15, 18, 19] and three were rated as moderate quality (5–7 stars) [16, 17, 20]. None of the included studies received a low-quality rating. Specific limitations were identified: in two studies the exposed cohort was potentially not representative of the target population (D1); [16, 17] in two studies there was no description of the of the response rate of participants or the characteristics of the responders or the non-responders (D3); [15, 18] in one study there was no mention of history of outcome in the controls (D4); [20] in two studies the outcome assessment methods were suboptimal (D6) [16, 17].

## Discussion

The study provides a detailed examination of the relationship between food insecurity and older adults, particularly in the context of extreme weather events. The screening process, which began with 1,709 records and narrowed to six eligible studies, was comprehensive but revealed several challenges. Firstly, the relatively total low number of retrieved studies highlights the novelty of the topic, as also confirmed from the publication date of the included articles. Moreover, the low number of included studies is also imputable to the paucity of studies that disaggregate data by age, highlight significant gaps in the existing literature, and prevent us from conducting a meta-analysis. Furthermore, the geographic concentration of studies, predominantly from the United States (three out of six), restricts the applicability of findings to other socio-cultural and environmental contexts.

Another important aspect identified in this systematic review is that the results obtained in individual studies are heterogeneous and seemingly contradictory. This high heterogeneity is supported by the great variability among studies. While some studies identified protective factors like social support and economic stability [16–18], others highlighted vulnerabilities due to extreme weather events, such as heatwaves and droughts [15, 19, 20]. This duality points to the heterogeneity in risk factors and resilience mechanisms, shaped by both individual and community-level attributes. Importantly, some studies relied on pre- and post-event comparisons [15–17, 19, 20], while others employed case-crossover methods [18], which could introduce methodological differences in how malnutrition and food insecurity are assessed. Furthermore, high variability was also assessed in terms of extreme weather events considered, follow-up, assessment methods and the definition of the outcome. Follow-up durations varied greatly, ranging from very short periods, such as 2 months for Arlappa et al., [15] to extended longitudinal designs spanning up to 15 [20] and 22 years [19]. Regarding assessment methods, there were significant differences: 3 studies of Clay et al. employed USDA criteria for food insecurity using surveys and validated food security screeners; Arlappa assessed undernutrition through BMI and chronic energy deficiency (CED) indices; and Mueller and Xu focused on undernutrition using caloric, fat, and protein intake measures and hospitalization data for protein-energy malnutrition [19, 20]. Similarly, the definition of outcomes varied, with three studies addressing food insecurity [16–18], while others examined undernutrition, either using BMI and CED [15] or as a broader clinical condition [19, 20]. Moreover, elderly populations are far from homogenous, with vulnerabilities shaped by a complex interplay of socioeconomic, gender, and geographic factors [22]. In this context, the characteristics of the studied populations add critical context to the findings. Elderly individuals included in the studies were often from socioeconomically disadvantaged backgrounds, such as those affected by hurricanes in the United States, droughts in India, or extreme heat exposure in China and Brazil [15–20]. These populations frequently exhibited gender imbalances (e.g., predominance of females in four studies), with women often at greater risk of food insecurity due to societal and economic vulnerabilities [23]. Moreover, socioeconomic factors such as income stability, employment status, and access to healthcare played significant roles in moderating food insecurity outcomes [24]. For instance, studies focusing on populations affected by extreme weather events (e.g., hurricanes) noted that homeowners with better income and social networks were less vulnerable [16–18]. Conversely, rural populations, particularly in India and China, faced higher risks due to limited resources, agricultural dependence, and geographic isolation [15, 19]. By synthesizing evidence from multiple countries and climate-related exposures (e.g., hurricanes, droughts, heatwaves), our study provides novel insights into the often-overlooked burden of climate-induced nutritional challenges in aging populations. These findings can inform more tailored public health strategies and policies aimed at mitigating the impact of climate change on the health and wellbeing of older adults.

### Potential biological mechanisms

Extreme weather events can significantly contribute to malnutrition and heightened food insecurity among older people through various biological and systemic mechanisms. Older subjects represent one of the most vulnerable population groups affected by climate change due to their physical, social, and economic conditions which limit their ability to adapt and access to sufficient and nutritious food resources [25]. Actually, older adults are particularly vulnerable due to age-related physiological changes, such as reduced appetite, altered nutrient absorption, and diminished metabolic efficiency, which increase their dependence on sufficient and nutritious food. Disruptions in food supply chains caused by extreme weather events, such as floods and droughts, often lead to spikes in food prices and limited availability of fresh, nutrient-dense products. This food product prices increment, makes healthy dietary patterns more challenging, in particular for low and middle-income individuals, including a growing number of elderly people [5]. An increase of food insecurity level can lead to unbalanced diets and malnutrition, increasing the risk of physical and cognitive impairments in older adults [26–28]. Extreme weather change events might play a significant role in increasing the risk or worsening malnutrition among older adults. Malnutrition in older adults can exacerbate their physical and cognitive decline, increasing susceptibility to chronic diseases and reducing their resilience to environmental stressors. Additionally, the intersection of food insecurity and climate-induced dietary imbalances further enhances health risks, creating a vicious cycle of vulnerability that undermines the overall well-being of aging populations.

### Implications for public health and policies

The findings of this study highlight the critical and urgent need for targeted public health interventions to address food insecurity, particularly among older populations who are disproportionately vulnerable [29, 30]. This vulnerability is exacerbated in regions prone to extreme weather events, where the intersection of environmental instability and socioeconomic challenges creates significant barriers to food security [5]. Addressing this issue requires a multi-faceted approach that considers both immediate needs and long-term resilience strategies.

One of the most pressing priorities is the strengthening of social safety nets and community support systems to protect older adults from economic instability [31]. Economic hardship is a key driver of food insecurity, particularly for elderly individuals with fixed or limited incomes. Public policies that enhance access to financial assistance, such as expanded pension programs or targeted food subsidies, can alleviate some of the economic pressures contributing to food insecurity [32, 33]. Additionally, community-based interventions, such as meal delivery services and food banks, can play a critical role in providing immediate relief while fostering social inclusion and support [34].

As highlighted in our findings, the three studies by Clay et al. demonstrate that protective factors, such as robust social support and effective health policies, play a crucial role in mitigating the impact of extreme weather events on older adults [16–18]. Social networks and a strong healthcare system act as essential buffers, reducing the negative effects of natural disasters [35]. Older adults, benefiting from stable incomes such as pensions, despite the general low income when compared to salary [36], might be less economically vulnerable compared to younger groups, who are more exposed to job losses and financial instability in the aftermath of disasters. Therefore, targeted interventions that strengthen economic and social support across all age groups remain vital to addressing these disparities, and prevent chronic diseases, effectively [37–39].

Another critical strategy involves integrating climate resilience into nutritional programs. Older populations are particularly susceptible to the health impacts of extreme weather events, such as heatwaves and droughts, which can disrupt food supply chains and access to essential resources [40]. Nutritional programs must therefore be designed with a focus on resilience, ensuring that they can operate effectively during and after such events. This might include diversifying food sources, supporting local agricultural initiatives, and creating robust distribution networks that prioritize access for vulnerable groups [41].

Enhancing disaster preparedness frameworks is also essential to address the unique challenges faced by older adults [42, 43]. Traditional disaster management strategies often overlook the specific needs of this demographic, particularly in terms of mobility, health, and dietary requirements [42]. By incorporating food security measures tailored to older adults into disaster preparedness plans, policymakers can ensure that this vulnerable group is not left behind during emergencies [42]. Such measures might include pre-positioning food supplies, creating emergency meal programs, and establishing rapid-response systems to address disruptions in food availability.

Finally, the study underscores the importance of advocating for climate mitigation policies that address the root causes of extreme weather events. Without significant efforts to curb climate change, the frequency and severity of events that disrupt food systems are likely to increase, exacerbating food insecurity for vulnerable populations [42, 44, 45]. Policymakers must therefore prioritize initiatives aimed at reducing greenhouse gas emissions, promoting sustainable agricultural practices, and investing in renewable energy [44]. These actions not only address the environmental drivers of food insecurity but also contribute to broader public health goals by creating more sustainable and resilient communities.

In conclusion, addressing food insecurity among older populations requires coordinated efforts across multiple sectors, integrating economic, environmental, and social strategies. By prioritizing these adaptive measures, public health policies can reduce the burden of food insecurity, improve health outcomes, and enhance the quality of life for elderly individuals in vulnerable regions.

### Future directions

Future research should address several key gaps identified in this study to advance our understanding of the relationship between food insecurity and malnutrition among older adults, particularly in the context of extreme weather events. First, there is a pressing need for longitudinal studies to track changes in food insecurity and malnutrition over time. The majority of existing studies rely on cross-sectional designs, which provide a snapshot of the problem but lack the ability to infer causality. Longitudinal research can help establish temporal relationships, enabling researchers to determine whether food insecurity precedes malnutrition or if other factors, such as health status or climatic events, mediate this relationship [46, 47]. These studies could also provide insights into how interventions or policy changes influence outcomes over extended periods.

Second, future studies should incorporate intersectional analyses to explore how the interaction of factors such as age, gender, ethnicity, and socioeconomic status influences nutritional outcomes [48]. Older adults are not a homogenous group, and their vulnerability to food insecurity and malnutrition varies depending on intersecting social determinants [8]. For example, women in certain cultural contexts may face additional barriers to accessing food, while racial and ethnic minorities in low-income settings often experience compounding disadvantages [23]. By adopting an intersectional lens, researchers can better identify which subgroups are most at risk and tailor interventions to their specific needs.

Third, addressing the gap in geographic variability is essential. Most current studies focus on high-income countries, yet food insecurity and malnutrition are disproportionately prevalent in low- and middle-income countries (LMICs). Regions such as sub-Saharan Africa, South Asia, and parts of Latin America remain underrepresented in the literature. Expanding research to include these areas is critical for understanding how diverse economic, social, and environmental contexts influence food security. These regions often face unique challenges, including limited infrastructure, climate vulnerability, and systemic inequalities, which must be considered when designing interventions [49–51].

Finally, there is an urgent need to adopt standardized measures for assessing food insecurity and malnutrition. Currently, studies employ a wide range of tools and definitions, making it challenging to compare findings across different contexts and populations. Uniform metrics would improve the consistency and reliability of data, enabling more robust meta-analyses and cross-country comparisons. Standardization could also facilitate the monitoring of trends over time, providing policymakers with actionable insights to inform interventions. Addressing these gaps through comprehensive, intersectional, and geographically inclusive research will significantly enhance our understanding of the complex dynamics of food insecurity and malnutrition. This, in turn, will support the development of targeted and effective policies to improve nutritional outcomes and mitigate the impact of food insecurity globally.

### Strengths and limitations

This study has several notable strengths that contribute to its significance and reliability. One of its key strengths lies in the comprehensive nature of the systematic review, which synthesizes data from multiple scientific databases. Additionally, the quality of the included studies was rigorously assessed using established frameworks such as the Newcastle-Ottawa Scale (NOS), which evaluates aspects like selection bias, comparability, and outcome assessment. This robust quality assessment ensures that the findings are based on reliable and methodologically sound evidence [52]. Furthermore, the inclusion of datasets encompassing both survey-based and clinical outcomes enriches the analysis, providing a more holistic understanding of the complex interplay between food insecurity and malnutrition.

However, the study also has several limitations that must be acknowledged. A major constraint is the high level of heterogeneity observed across the included studies, which restricts the generalizability of the findings. This variability likely arises from differences in study populations, contexts, and methodological designs, making it challenging to synthesize results into universally applicable conclusions. In light of this heterogeneity, we did not perform a meta-analysis opting instead for a narrative synthesis of the findings. This approach allowed us to qualitatively explore patterns, contextual differences, and potential risk or protective factors, while avoiding inappropriate pooling of methodologically diverse data. This approach prevents us to under or overestimated the results and therefore conclusions. Another potential limitation of this review is the restriction to studies published in English. However, no potential eligible studies have been excluded to the language issue. Methodological differences, such as the use of varied measures to assess the outcome and different outcome definitions, further complicate comparisons across studies, adding an additional layer of complexity to the interpretation of the results. Taken together, while this study offers valuable insights into the relationship between food insecurity and malnutrition, the limitations underscore the need for careful interpretation of the findings and highlight areas for improvement in future research.

## Conclusions

This systematic review reveals that extreme weather events may significantly affect food security and nutritional outcomes among elderly populations. While certain studies identified protective factors, such as social support and economic stability, others highlighted vulnerabilities, particularly in regions with pronounced climatic stressors like hurricanes, droughts, and heatwaves.

Despite findings underscore the complex interplay between environmental and socioeconomic determinants of food insecurity and malnutrition, future studies are needed in order to confirm our preliminary results. Vulnerable populations, particularly those in socioeconomically disadvantaged settings, are disproportionately impacted by disruptions in food supply and access. Our findings underscore the urgent need for integrated public health strategies to address food insecurity among older adults, particularly in the face of extreme climate events. Effective interventions should include the strengthening of social safety nets, economic support measures, and community-based services that ensure access to nutritious food. Equally important is the integration of climate resilience into nutritional programs and disaster preparedness plans tailored to the specific needs of older populations. Finally, long-term solutions must involve climate change mitigation policies aimed at reducing environmental disruptions to food systems. A coordinated, multisectoral approach is essential to protecting the health and wellbeing of aging populations in an increasingly unstable climate.

Addressing extreme weather events challenges will contribute to reducing the burden of malnutrition and food insecurity among the elderly, improving their health and quality of life in an era of increasing climatic instability.

## Data Availability

No datasets were generated or analysed during the current study.
